# Development and Validation of AAV Capsids Separation on Specimen Columns for Reproducibility Evaluation of Large‐Scale Chromatographic Monoliths

**DOI:** 10.1002/jssc.70114

**Published:** 2025-03-17

**Authors:** Rok Miklavčič, Tina Simčič, Sara Rotar, Polona Komel, Rok Žigon, Dona Pavlovič, Ines Bergoč, Domen Ipavec, Ana Simčič Zuljan, Ažbe Žnidaršič, Dolores Kukanja, Jana Vidič, Aleš Štrancar, Urh Černigoj

**Affiliations:** ^1^ Sartorius BIA Separations d.o.o. Ajdovščina Slovenia; ^2^ Faculty of Medicine University of Ljubljana Ljubljana Slovenia

**Keywords:** batch reproducibility, down‐scaling, gene therapy, preparative chromatography, scale‐up

## Abstract

One of the key challenges in adeno‐associated virus (AAV) viral vector manufacturing is the effective and consistent separation of full (F) AAV capsids from undesired non‐functional (empty = E, partially filled, etc.) capsids. Typically, at least one chromatography step is used for this purpose in AAV manufacturing. Due to the complexity of viral capsids separation, even a small change in the chromatographic process is reflected in unreproducible results. One solution for robust polishing of full AAV capsids is the highly reproducible (HR) design of chromatographic columns used in this step. Implementation of such columns requires the development of control tests, which efficiently predict column performance for AAV separation. In this paper, the methodology for reproducible separation of empty and full recombinant AAV2/8 (E/F rAAV2/8) capsids was defined using quaternary amine (QA) chromatographic monoliths in a linear potassium chloride (KCl) gradient. The scalability of the procedure was experimentally confirmed on 1, 80, and 800 mL CIMmultus QA columns, where empty capsids eluted at a KCl concentration range of 89.4–91.4 mM. A sampling of the monolith material from the 800 mL CIMmultus QA column and testing it for E/F rAAV2/8 capsid separation in the form of a 200 µL column resulted in a highly comparable elution pattern as obtained with the parent 800 mL column. The principle of sampling material by cutting the parent monolith, packing it in 200 µL columns (specimens) and testing them for E/F rAAV2/8 capsid separation was further developed to demonstrate intra‐column homogeneity; batch‐to‐batch homogeneity; and scalability of CIM QA monoliths. Finally, specimens testing using a validated E/F rAAV2/8 separation method was used to monitor 28 CIMmultus QA production batches (bed volumes between 1 and 8000 mL). E rAAV2/8 capsids eluted at KCl concentration between 89.3 and 95.3 mM for 28 batches, paving the way for commercialization of highly reproducible preparative QA chromatographic monoliths (CIMmultus QA HR).

**Abbreviations**
AAV: adeno‐associated virus
AEX: anion exchange
BTP: 1,3‐bis(tris(hydroxymethyl)methylamino)propane
CIM: Convective Interaction Media
CV: column volume
dPCR: digital polymerase chain reaction
E: empty, partially filled, misfolded (non‐functional) capsids
E/F AAV: empty and full adeno‐associated virus capsids
F: full (functional) capsids
HPG: high‐pressure gradient
HR: highly reproducible
ITR: inverted terminal repeats
LC: liquid chromatography
LPG: low‐pressure gradient
MALS: multi angle light scattering
MWCO: molecular weight cut‐off
MWD: multi‐wavelength UV–Vis detector
PES: polyethersulfone
pI: isoelectric point
QA: quaternary amine
rAAV2/8: recombinant adeno‐associated virus, serotype 8
*R*_full/empty_
: resolution between peaks corresponding to full and empty capsids
RSD: relative standard deviation
TFF: tangential flow filtration
TRIS: tris(hydroxymethyl)aminomethane
vg: viral genomes
vp: virus particles

## 1 Introduction

Recombinant adeno‐associated viral (AAV) vectors have emerged as very promising gene delivery tools and have already been used in more than 200 ongoing pipeline drug developments and eight approved products on the market [1, 2]. Ensuring the purity of in vivo administered AAVs for gene therapy is paramount for their efficacy and safety [3]. Many AAV downstream processes have already been developed and are used in the clinical manufacturing of AAV [4, 5] employing at least one ultracentrifugation [6, 7] or a chromatography step. Although large‐scale ultracentrifugation has been industrially demonstrated in AAV vector purification [8], the capital investment associated with this technology makes it difficult to implement in small‐size facilities. Linear scalability in the manufacturing process is more easily achieved by chromatography, which often incorporates a primary capture step employing affinity [9, 10], cation‐exchange [11], or preferential exclusion [12] chromatography, followed by a secondary, polishing step, typically employing anion‐exchange (AEX) chromatography for separation of full (F) capsids from empty (E), partially filled, misfolded, and multimeric capsids. E/F AAV separation is based on approximately 0.5 pH unit difference in the isoelectric point (pI) between F and E capsids and the separation is usually performed in an ascending linear salt gradient [13]. Small differences in surface charge distribution and density between E and F AAV capsids lead to low chromatographic selectivity, and consequently, baseline separation is challenging to achieve [14]. The control and reproducibility of industrial processes become extremely vulnerable toward small variations in equipment specifications, which can include cross‐scale differences in the volumetric accuracy of pumps, proportioning accuracy of the gradient mixing system, and overall fluidics architecture [15]. In addition, chromatographic method‐based sources of variations include the consistency of mobile phase composition and its aging, amount of dissolved air variability in the chromatography media, column equilibration as well as separation temperature [16]. These parameters negatively influence the robustness of process scale‐down and/or scale‐up [17] as well as the robustness of E/F AAV downstream purification process.

Different attempts to improve the E/F AAV separation have been reported in the literature. Studies have highlighted the ineffectiveness of improving baseline E/F AAV separation with variables such as buffer content (e.g., BTP, AMPD, AMPSO, CAPSO), salt types (e.g., NaCl, KCl, quaternary amines, Na_2_SO_4_, Mg^2+^ salts) [18, 19, 20, 21] and employing dual salt elution gradients [22, 23]. Researchers wanted to overcome poor selectivity by replacing a linear salt gradient by the application of more complex gradient formations, such as the implementation of isocratic washes [23]; a two‐step step gradient [24]; weak partitioning multi‐column AEX chromatography [25]; AEX chromatography with continuous recycling and accumulation [26], however, the robustness of chromatographic separation remains restricted by poor E/F selectivity. The separation power of AEX chromatography could be improved using novel chromatographic ligands enabling additional analyte‐chromatographic surface interactions—hydrogen bonding, metal affinity, or hydrophobic properties. Recently, CIMmultus PrimaT and CIMmultus PrimaS monoliths, both operating through multimodal AEX ligands, were evaluated for E/F AAV separation [27, 28]. Despite the improvements in E/F resolution, baseline separation or considerably improved robustness has not been achieved. Only one report of considerably improved E/F AAV8 and AAV5 separation using Convective Interaction Media (CIM) quaternary amine (QA) columns has been reported, but only tested on a limited number of samples so far [29].

The failure to considerably increase the selectivity of E/F separation calls for improved control over chromatographic media. Convection‐aided chromatography media, such as monoliths and membranes, are often preferred for virus separation due to very low mass transfer resistance and monoliths in particular for their higher resolution power at any flow rate [30, 31]. However, due to the nature of the production process—the fact that each monolith could be considered its own batch, lot‐to‐lot reproducibility of the manufacturing process requires careful attention. For majority of biotech applications, sufficient lot‐to‐lot production consistency has already been demonstrated [32, 33, 34], but there are studies claiming that inconsistent AAV elution profiles were obtained for E/F AAV separation with CIM QA monoliths using a platform step gradient elution method [35], suggesting that columns intended for this application require narrower release specifications, which should be able to differentiate between slight column differences, particularly for large‐scale monolith batches [36]. Establishing a QC method employing the E/F AAV separation as a release criterion for chromatographic columns is a logical step toward providing highly reproducible (HR) columns. However, due to the structure of a chromatographic monolith, conventional methods for resin sampling and characterization cannot be applied to monoliths. The prerequisite for successful testing of batch‐to‐batch reproducibility is a so‐called “specimen column”—a representative small scale‐chromatographic column, obtained from a parental large‐scale monolith column by mechanical excision. It is preferred for manufacturing that chromatographic steps maintain the same elution condition (conductivity or pH) of target molecule regardless of column/process scale, because this enables a relatively easy transfer to step gradient methods and simplification of chromatography. To successfully scale‐up a linear gradient while maintaining the salt concentration at elution (iso‐conductivity scale up), it is crucial to maintain a constant gradient slope [37, 38], defined as normalized gradient slope (GH), see Equation (1). When changing column scales, GH can be held constant by appropriately adjusting the flow rate and/or gradient time.

(1) $$\mathrm{GH}=\frac{I_{f}-I_{0}}{V_{g}}\times \left(V_{t}-V_{0}\right)$$

where *I*_f_ and *I*_0_ in stand for final and initial salt concentrations, respectively. *V*_g_ is gradient volume while *V*_t_ is the total bed volume and *V*_0_ is the column void volume [37].

Here, we report the development of a testing procedure for evaluating the homogeneity and scalability of large‐scale CIM QA monoliths. We establish a protocol for obtaining representative specimen columns from parental large‐scale monoliths, followed by the development and validation of a chromatographic test method based on E/F rAAV2/8 capsid separation. The scalability of the chromatographic test is verified by reproducing the rAAV2/8 capsids separation on a 0.2 mL specimen and the corresponding 800 mL parental column. The new tools enable us to demonstrate the scalability of the manufacturing process of the commercial CIMmultus QA monolithic column line across the entire range of production scale (1–8000 mL).

## 2 Materials and Methods

### 2.1 Chemicals and Reagents

All buffers were freshly prepared with European Pharmacopoeia‐grade purified H_2_O and analytical‐grade reagents. Buffer solutions were filtered through a 0.2 µm PES filter (Nalgene Rapid‐Flow, Thermo Fisher Scientific, Waltham, MA, USA). Uracil, 1,3‐bis(tris(hydroxymethyl)methylamino)propane (BTP), magnesium chloride hexahydrate (MgCl_2_·6H_2_O), Poloxamer 188, D‐Saccharose, potassium chloride (KCl), and 1 M hydrochloric acid (Titripur) were obtained by Merck (Darmstadt, Germany). Tris(hydroxymethyl)aminomethane (TRIS), Tween 20 and sodium hydroxide (NaOH) were obtained by Sigma–Aldrich (St. Louis, MO, USA). Sodium chloride (NaCl) was obtained by Honeywell (Charlotte, NC, USA). Ethanol was purchased from Pharmachem (Ljubljana, Slovenia).

### 2.2 rAAV2/8 Production, Purification and Preparation of Standard rAAV2/8 Solution

rAAV2/8 was generated through triple transfection of suspension HEK293 cell line in chemically defined media. Rep2‐Cap8 and Helper plasmids were used together with cis construct containing GFP expression cassette flanked by inverted terminal repeats (ITRs) regions from AAV2. Plasmids were combined in molar ratio 1:1:1 and transfected to cells using PEI MAX transfection reagent from Polysciences (Warrington, PA, USA). Transfection was performed in 5 L stirred‐tank Biostat B‐DCU bioreactor (Sartorius, Goettingen, Germany) in fed‐batch mode. Cell lysis was performed 48 h post‐transfection by adding Tween 20 detergent directly into bioreactor. Material was harvested and frozen at −80°C until further use.

The lysate was clarified by train of filters connected in series using Sartopure GF+ 1.2 µm (Sartorius) followed by Sartopore 2 0.45 µm (Sartorius). Clarified harvest was concentrated and pre‐treated by tangential flow filtration (TFF) Sartocon Slice holder using Hydrosart 300 kDa cassettes (Sartorius) including within‐system salt‐tolerant nuclease digestion using Saltonase (Blirt, Gdańsk, Poland). In the next step, pH of the solution was adjusted to meet the binding conditions, clarified again by Sartopore 2 0.45 µm (Sartorius) and captured using cation exchange chromatography with CIMmultus SO3 column with 2 µm channel size (Sartorius BIA Separations, Ajdovščina, Slovenia). For the polishing step, SO3 eluate was diluted, further purified, and enriched for full capsids using anion exchange CIMmultus QA column with 2 µm channel size (Sartorius BIA Separations), loaded at 1.25E13 viral genomes (vg) per 1 mL of column. rAAV2/8 capsids were eluted by applying a linear ascending salt gradient and performed using ÄKTA Avant 150 system (Cytiva, Marlborough, MA, USA) at room temperature. Empty and full capsid fractions were collected separately. After chromatography, each main fraction was buffer exchanged for 5 to 6 diavolumes in formulation buffer (20 mM TRIS, 150 mM NaCl, 2 mM MgCl_2_, 0.01% poloxamer 188, pH 7.5) using a Vivaspin 20 centrifugal concentrator with 100 000 molecular weight cut‐off (MWCO) polyethersulfone (PES) membrane (Sartorius).

The final rAAV2/8 standard sample was prepared by mixing fractions of purified empty and full rAAV2/8 capsids to achieve a final E/F ratio of approximately 1:1. The standard sample was aliquoted and frozen at under −75°C. The final composition of a standard matrix was 20 mM TRIS, 150 mM NaCl, 2 mM MgCl_2_, 0.01% poloxamer 188, pH 7.5.

### 2.3 Analytical Methods Used for In‐Process Control Monitoring During Preparation of AAV Standards

#### 2.3.1 Digital Polymerase Chain Reaction (dPCR)

dPCR QIAcuity One System (Qiagen, Hilden, Germany) method was used to evaluate rAAV2/8 vector genome titer. Samples were treated with DNaseI (NEB, Ipswich, MA, USA) to digest non‐encapsidated DNA. Serial dilutions (10×) of samples were prepared and samples were incubated at 95°C for 10 min to open AAV capsids. Dilutions that fit the dynamic range of dPCR were selected and pipetted onto 96‐well nanoplate (Qiagen), followed by a PCR reaction and recording. Selected primers and probes for the PCR reaction were designed to align onto the eGFP target. Detail design, analysis, and quantification were done using the Qiagen dPCR user manual.

#### 2.3.2 PATfix E/F AAV Analytics

For both quantitative and qualitative assessment of E to F AAV capsids ratio, an anion exchanging liquid chromatography (LC) method (PATfix‐AEX) employing a CIMac AAV full/empty‐0.1 Analytical Column (1.3 µm) on an analytical PATfix low pressure‐gradient (LPG) system (Sartorius BIA Separations) was used. Buffer A was 20 mM BTP, 2 mM MgCl_2_, 1% sorbitol, 0.1% poloxamer 188, pH 9.0 and buffer B was 20 mM BTP, 400 mM NaCl, 2 mM MgCl_2_, 1% sorbitol, 0.1% poloxamer 188, pH 9.0. 500 µL of diluted sample was injected onto the column, followed by 1 min wash with buffer A and separation of E/F AAV capsids performed in a linear gradient from 100% buffer A to 100% buffer B over 10 min at a flow rate of 1 mL/min. Data was analyzed using PATfix v2.0 software (Sartorius BIA Separations). The percentage of full AAV capsids was determined by integrating the fluorescence signal.

### 2.4 Chromatographic Experiments on CIMmultus QA Column Line

#### 2.4.1 Chromatographic Columns and Preparation of 0.2 mL Specimens

Experiments were carried out on CIMmultus QA 1, 80, 800 mL (QA, strong AEX) chromatographic monolithic columns with channel diameter of 2.0 µm (Sartorius BIA Separations).

**FIGURE 1:**
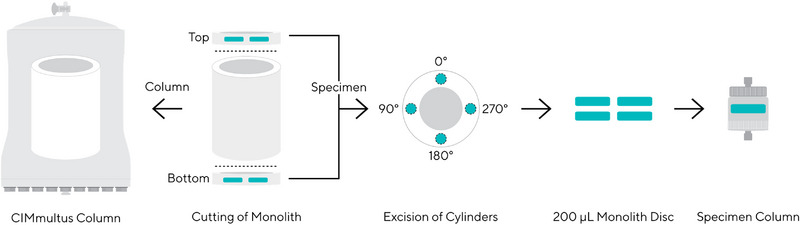
Schematic representation of obtaining parental columns and specimen columns from the initial monolith. Dashed lines represent cutting or drilling.

We define “specimen” as a small chromatographic column with 0.2 mL bed volume, sampled from the bulk single‐piece monolith during production by mechanical excision. The excision procedure was as follows: after polymerization and functionalization of 80, 800, and 8000 mL tubular QA monoliths, slices (10 mm height) from top and bottom part of the tube were excised. The remaining central tube was packed as a parental column in a standard CIMmultus housing (80, 800, and 8000 mL size), while cylinders with a diameter of 7.5 mm were drilled from top and bottom slices at different positions in perpendicular directions (see Figure 1). The cylinders were finally packed as disc‐shaped monolithic columns with a diameter of 7.5 mm and a height of 4.2 mm in a dedicated chromatographic housing with axial flow distribution.

For intracolumn homogeneity evaluations, the parental tubes were additionally sectioned to five vertical (A, B, C, D, and E) slices, as shown in Figure S1. Each letter represents a defined height of the parental monolith, A being at the bottom part of the tube and E being at the top. The specimen disc was taken from each slice at 0°, 90°, 180°, and 270° horizontal positions, where each number represents an angle, at which the specimen disc was cut (see Figure 1).

#### 2.4.2 Chromatographic Systems

Chromatographic experiments with 0.2 mL specimen columns and 1 mL columns were performed using a PATfix LC system composed of a high pressure‐gradient (HPG) pump, a multi‐wavelength UV–Vis detector (MWD) with 10 mm flow cell path length, a conductometer, a pH monitor and a fluorescence detector (Shimadzu, Kyoto, Japan), additionally equipped with a column thermostat (Shimadzu). Where temperature was controlled, columns were kept inside the column thermostat and inflowing buffer temperature was maintained by a preheating block consisting of a stainless‐steel tubing (length 1 m, ID 0.17 mm) wrapped around a proprietary stainless‐steel block, which was also placed inside the column thermostat in front of the column. UV absorbance was monitored at 260 and 280 nm for AAV separation or at 200 nm for ionic capacity determination. For AAV separation, intrinsic protein fluorescence was induced with an excitation wavelength of 280 nm and monitored at an emission wavelength of 348 nm. PATfix v2.0 software was used for instrument control and data analysis.

The chromatographic scale up experiments using 1 mL column were performed on an ÄKTA pure 150 M (Cytiva), composed of a binary pump, a MWD (10 mm flow cell path length) and an integrated conductometer. An additional conductometer (Knauer, Berlin, Germany) and a fluorescence detector (Shimadzu) were placed after the integrated conductometer to monitor the relevant signals on the same set of equipment. UNICORN (Cytiva) software was used for instrument control and data acquisition.

During scale‐up 80 and 800 mL columns were evaluated on an Azura prep HPG system (Knauer), composed of a binary pump, a single wavelength UV–Vis detector (2 mm flow cell path length), and an integrated conductometer. After the conductometer, the flow was split into two lines with a PEEK tee (VICI Jour, Schenkon, Switzerland) and adjusted to 1 mL/min in the line, where the same additional conductometer (Knauer) and fluorescence detector (Shimadzu) as with 1 mL column were placed (Figure S2). ClarityChrom (Knauer) was used for instrument control, while PATfix v2.0 software was used for data analysis.

#### 2.4.3 Ionic Capacity Testing of CIMmultus QA 1 mL Columns

The ionic capacity measurements on CIMmultus QA 1 mL columns were performed by using two sodium phosphate buffer solutions having the same pH, but varying in ionic strength [36]. The column was first equilibrated with 0.5 M sodium phosphate, pH 6.5, until presaturation of absorbance at 200 nm and pH was reached. To form a pH transient, buffer was switched from 0.5 M sodium phosphate, pH 6.5 to 10 mM sodium phosphate, pH 6.5 at 5 mL/min. The experiment was completed when the absorbance and the pH of the effluent reached the absorbance and the pH of the solution at the column inlet. The time between switching the mobile phases and reaching 50% absorbance of the “breakthrough” was determined. The ionic capacity *K* was calculated according to Equation (2).

(2) $$K=\frac{\Delta t_{\mathrm{pH}\left(50\%\right)}\times F_{V}\times c_{\mathrm{MPB}}}{V_{c}}\;$$

where Δ*t*_pH(50%)_ represents the duration of the pH transient time difference at 50% of the pH shift, *F*_V_ is volumetric flow rate (mL/min), *c*_MPB_ stands for phosphate concentration in the low conductivity buffer (10 mM), and *V*_c_ is column volume (mL).

#### 2.4.4 Chromatographic Separation of E/F rAAV2/8 Capsids on CIMmultus QA 1 mL Columns

CIMmultus QA 1 mL columns were evaluated for rAAV2/8 E/F separation in ascending KCl gradient at a working flow rate of 2 mL/min (2 CV/min). Buffer A was composed of 25 mM BTP, 51 mM KCl, 2 mM MgCl_2_, 1% w/w D‐saccharose, 0.1% w/w poloxamer 188, pH 9.0, while Buffer B was composed of 25 mM BTP, 153 mM KCl, 2 mM MgCl_2_, 1% w/w D‐saccharose, 0.1% w/w poloxamer 188, pH 9.0. Conductivities of buffers A and B, measured with InLab 731 conductivity probe (Mettler Toledo, Greifensee, Switzerland) at a reference temperature of 25 C, were 7.90–8.10 mS/cm and 19.80–20.20 mS/cm, respectively.

Prior to evaluation, columns were rinsed with 10 CV of water and cleaned with 10 CV of 0.1 M NaOH, 2 M NaCl solution. Immediately after, the columns were washed with 30 CV of 100 mM BTP, 1 M KCl, pH 9.0. Next, the columns were washed with 10 CV of water and equilibrated with 10 CV buffer A, 10 CV buffer B, and 20 CV buffer A.

During AAV separation, temperature was fixed at 23 ± 1°C with a column thermostat, as described in Section 2.4.2. Columns were in the thermostat for at least 30 min prior to analysis.

rAAV2/8 test sample was prepared in MPA and composed of non‐binding tracer (uracil) and E and F rAAV2/8 capsids with an E/F capsid ratio of approximately 1:1. For column evaluation, 3E14 virus particles (vp) of rAAV2/8 test sample was prepared, aliquoted in ready‐to‐inject format and stored at −80°C. Prior to evaluation, a fresh aliquot of rAAV2/8 test sample was thawed, vortexed and then used immediately. A single 250 µL injection used for evaluation of 1 mL columns contained approximately 5 µg of uracil (5 µg of uracil per mL of monolith) and 2.5E10 vg of rAAV2/8 full capsids (2.5E10 vg of rAAV2/8 per mL of monolith).

The separation method is composed of the following steps: 0.5 min wash with 100% buffer A, 15 min linear gradient from 100% buffer A to 100% buffer B, 2.5 min hold at 100% buffer B, and 7 min re‐equilibration with 100% buffer A.

To compare elution conditions of E/F rAAV2/8 capsids between analyses, a calculated parameter was implemented—normalized KCl concentration at elution of E (*[KCl]*_empty_) or F (*[KCl]*_full_) capsids. *[KCl]*_empty_ and *[KCl]*_full_ were calculated with Equation (3) by normalizing the chromatographic conductivity at empty/full capsid elution (*σ*_empty/full_) to chromatographic conductivity values of buffer A (*σ*_buffer A_) and buffer B (*σ*_buffer B_) and converting this to nominal KCl concentration.

(3) $${\left[KCl\right]}_{\mathrm{empty}/\mathrm{full}}=\frac{\sigma_{\mathrm{empty}/\mathrm{full}\;}-\sigma_{\mathrm{buffer}\;A}}{\sigma_{\mathrm{buffer}\;B}-\;\sigma_{\mathrm{buffer}\;A}}\;\times f1+f2\;$$

where *[KCl]*_empty/full_ is the normalized KCl concentration at empty/full capsid elution (mM). *σ*_empty/full_, *σ*_buffer A_, and *σ*_buffer B_ are conductivities (in mS/cm) at empty/full capsids’ elution, 100% buffer A and 100% buffer B, respectively, recorded with the conductometer on the system. *f1* is 102 mM (difference in nominal *[KCl]* between buffer B and buffer A) and *f2* is 51 mM (nominal *[KCl]* in buffer A).

Resolution between peaks corresponding to full and empty capsids (*R*_full/empty_) was calculated according to the European Pharmacopoeia using Equation (4), integrated in the PATfix v2 software.

(4) $$R_{\mathrm{full}/\mathrm{empty}}=1.18\times \frac{t_{\mathrm{full}}-t_{\mathrm{empty}}}{w_{\mathrm{full}}+w_{\mathrm{empty}}}$$

where *t*_full_ and *t*_empty_ are the retention times of full and empty capsid peaks, while *w*_full_ and *w*_empty_ are their width at half height. Signals from the fluorescence detector were used.

#### 2.4.5 rAAV2/8 Separation on 0.2 mL Specimen Monolithic Columns and Method Validation

The method of separating E/F rAAV2/8 capsids using 0.2 mL specimen columns resembles the method used for evaluation of 1 mL columns (Section 2.4.4) with the following differences: flow rate of 1 mL/min (5 CV/min) was used and 100 µL of sample was injected, which contained approximately 1 µg of uracil (5 µg of uracil per mL of monolith) and 5E9 vg of rAAV2/8 full capsids (2.5E10 vg of rAAV2/8 per mL of monolith). The separation method comprised the following steps: 0.5 min wash with 100% buffer A, 6 min linear gradient from 100% buffer A to 100% buffer B, 2.5 min hold at 100% buffer B and 5 min re‐equilibration with 100% buffer A.

rAAV2/8 separation method was validated using one specimen obtained from 800 mL monolith. The following chromatographic parameters were evaluated: (1) inter‐day repeatability, (2) intra‐day repeatability, and (3) repeatability using three different PATfix systems with the same hardware composition.

#### 2.4.6 Scaling up E/F rAAV2/8 Separation to the CIMmultus QA 80 and 800 mL Bed Volume

Scalability was evaluated using CIMmultus QA column line (1, 80, and 800 mL columns) as well as 0.2 mL specimen columns. Column preparation and the chromatographic method for separation of E/F rAAV2/8 capsids were identical as described in Section 2.4.4 with the following differences. The flow rate for column preparation and separation was adjusted to 1 CV/min on all column scales. The gradient was scaled up from 1 mL to 80 and 800 mL column sizes using iso‐conductivity approach [37], that is, by maintaining a constant gradient length of 30 CV. For this experiment, new rAAV2/8 sample was prepared and loaded to the columns in the amount of 5.3E10 vg per mL of the monolith in 1 CV (tryptophan was not added to the sample). The temperature was monitored during separation. The incoming buffer temperature was in the range of 23 ± 1°C in all experiments.

After separation of rAAV2/8, the 800 mL column was cut into three vertical slices (height A, C, and E—see Figure S1), from which specimens were obtained according to the procedure described in Section 2.4.1. The specimen columns were again evaluated for rAAV2/8 separation, together with control specimens from above and below the part of large‐scale monolith, which was packed in the 800 mL column. The specimens were evaluated under the conditions described in Section 2.4.5, but using the same rAAV2/8 sample (and sample loading at 5.3E10 vg per mL of monolith in 1 CV) and buffers, that were used to evaluate the 800 mL column.

## 3 Results and Discussion

The first purpose of the study was to identify and implement highly controlled chromatographic conditions for separation of E/F AAV capsids that can be easily adapted for any size of the chromatographic monolith. Such conditions are prerequisites for the development of a tool for monitoring the variability and the scalability of chromatographic monoliths for E/F AAV separation from 1 mL up to 8000 mL column size. The method should be relatively simple, applicable to every monolithic column size and should enable sorting the produced cGMP chromatographic columns according to the differences in E/F AAV capsid separation.

### 3.1 Chromatographic Method for Separation of E/F rAAV2/8 Capsids

**FIGURE 2:**
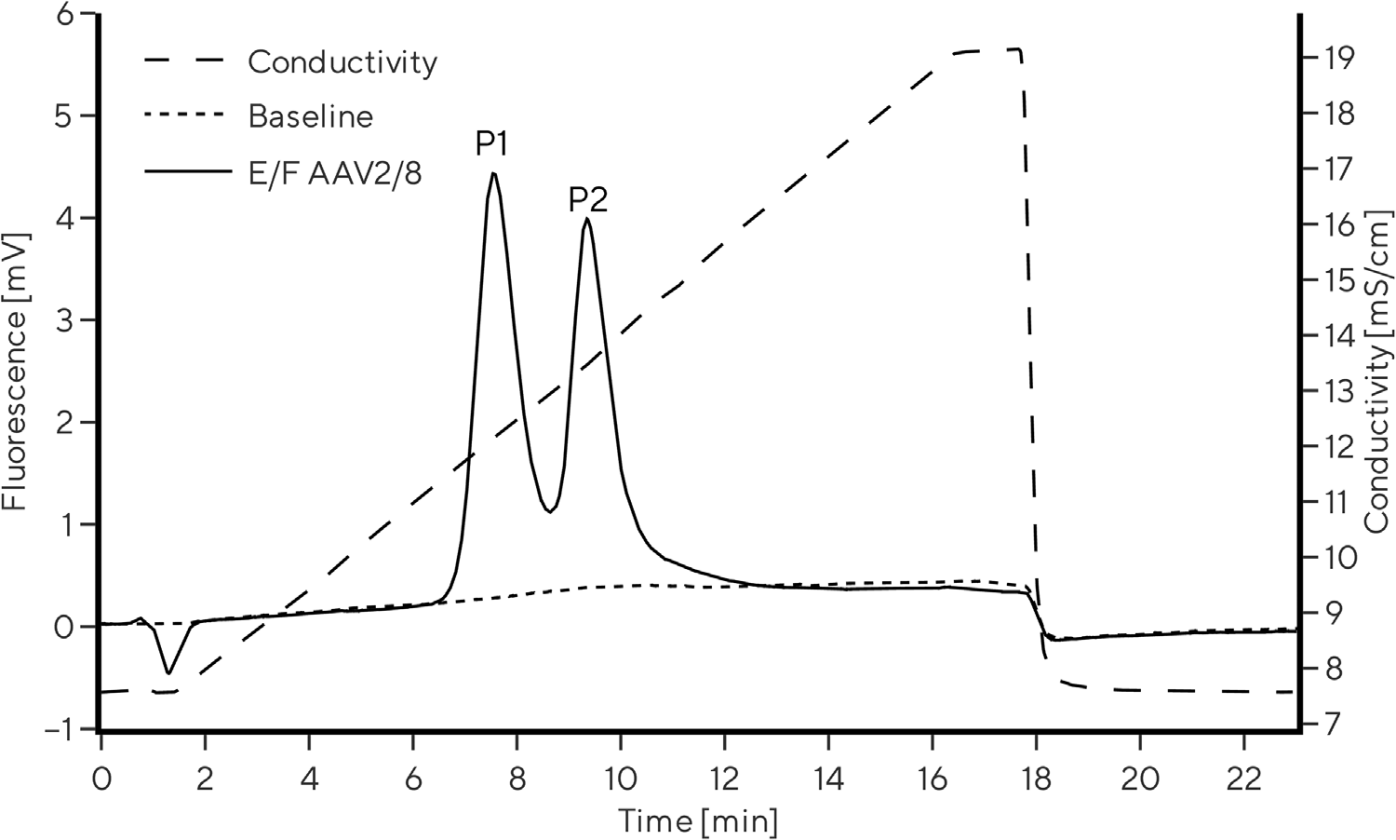
A representative chromatogram of analytical E/F rAAV2/8 capsid separation using CIMmultus QA 1 mL column in ascending KCl gradient. Fluorescence and conductivity traces are shown. First peak (P1) corresponds to empty rAAV2/8 capsids and the second peak (P2) corresponds to full rAAV2/8 capsids.

First, a robust and reliable chromatographic method for separation of E/F rAAV2/8 capsids was needed as a reference chromatographic method, which would enable the evaluation and comparison of CIMmultus QA columns properties from different batches and sizes. The focus was not on the peak resolution, but on the method robustness and easy‐to‐implement parameters. CIMmultus QA 1 mL columns, the smallest radial column of the commercially available preparative CIM columns, were used for this purpose. Control of the following chromatographic parameters was found crucial for reliable separation of E/F rAAV2/8 capsids: column temperature, mobile phase composition, column conditioning, void volume of the chromatographic path, sample preparation, and amount of loaded sample [39]. The chromatographic method, as it is described in Section 2, considers all influences described above. E/F rAAV2/8 capsid separation is achieved in linear ascending KCl gradient (from 51 to 153 mM KCl) with a gradient slope of 3.4 mM KCl per CV of the monolithic column. A typical chromatogram obtained with CIMmultus QA 1 mL column is shown in Figure 2 with two main peaks—corresponding to empty (P1) and full rAAV2/8 capsids (P2). Intrinsic protein fluorescence was used for detection due to higher sensitivity compared to UV signal, and consequently, lower consumption of rAAV2/8 standard sample for a single test. Moreover, only FLD signal was proven to portray the actual ratio between empty and full AAV capsids without the need of mass or molar extinction coefficient correction factor [40]. A premixed, aliquoted, and frozen standard rAAV2/8 test solution was used as a probe sample to avoid negative effects on the analysis results due to the sample preparation and its stability (see Section 2).

The precision and accuracy of monitored conductivity values from LC detectors is ± 2%, which is not sufficiently accurate to monitor E/F AAV elution conditions, where ± 0.4 mS/cm already represents a significant difference. Therefore, for comparison of elution conditions of empty and full capsids between chromatographic analyses, a calculated parameter was implemented—normalized KCl concentration at elution of empty or full capsids (*[KCl]*_empty/full_). This parameter correlates the AAV retention times and measured conductivities to nominal KCl concentration in buffers A and B (see Equation 3).

**FIGURE 3:**
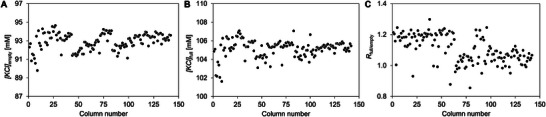
Results of E/F rAAV2/8 separation on 142 CIMmultus QA 1 mL columns. Scattering of *[KCl]*_empty_ (A), *[KCl]*_full_ (B), and *R*_full/empty_ (C) is demonstrated.

For 142 tested CIMmultus QA 1 mL columns, *[KCl]*_empty_, *[KCl]*_full_ and *R*_full/empty_ were in the ranges of 91.1–94.2 mM (with 0.8% RSD), 103.2–107.1 mM (with 0.6% RSD) and 0.85–1.25 (with 7.5% RSD), respectively (Figure 3). The E/F resolution was scattering 10 times more than the scattering of eluting salt concentration, and in addition, higher resolution did not correlate with higher or lower elution point of empty or full capsids. Moreover, since AAV capsid separation on QA columns is performed in linear or step ascending salt gradient, constant salt concentration at AAV elution is the crucial parameter from a robust preparative chromatographic process perspective. Therefore, *[KCl]*_empty_ was used as the main chromatographic parameter representing AAV elution in the following experiments.

### 3.2 Separation of E/F rAAV2/8 Capsids on Large‐Scale Chromatographic Columns

With a robust and reproducible chromatographic method in hand, we evaluated the scale‐to‐scale reproducibility of CIMmultus QA columns by separating E/F rAAV2/8 capsids using 1, 80, and 800 mL columns. The chromatographic method used in Section 3.1 for 1 mL columns was adjusted and scaled up to 80 and 800 mL column sizes using the iso‐conductivity scale‐up approach [37], thus expecting similar elution conductivities. Due to a very low rAAV2/8 load, intrinsic protein fluorescence was monitored to detect elution. Fluorescence and conductivity were monitored with the same set of detectors at a fixed flow rate of 1 mL/min to eliminate the effect of hardware variability and detector response time at different flow rates. Flow rate reduction for 80 and 800 mL columns was achieved by a flow splitting tee, as described in Figure S2.

**FIGURE 4:**
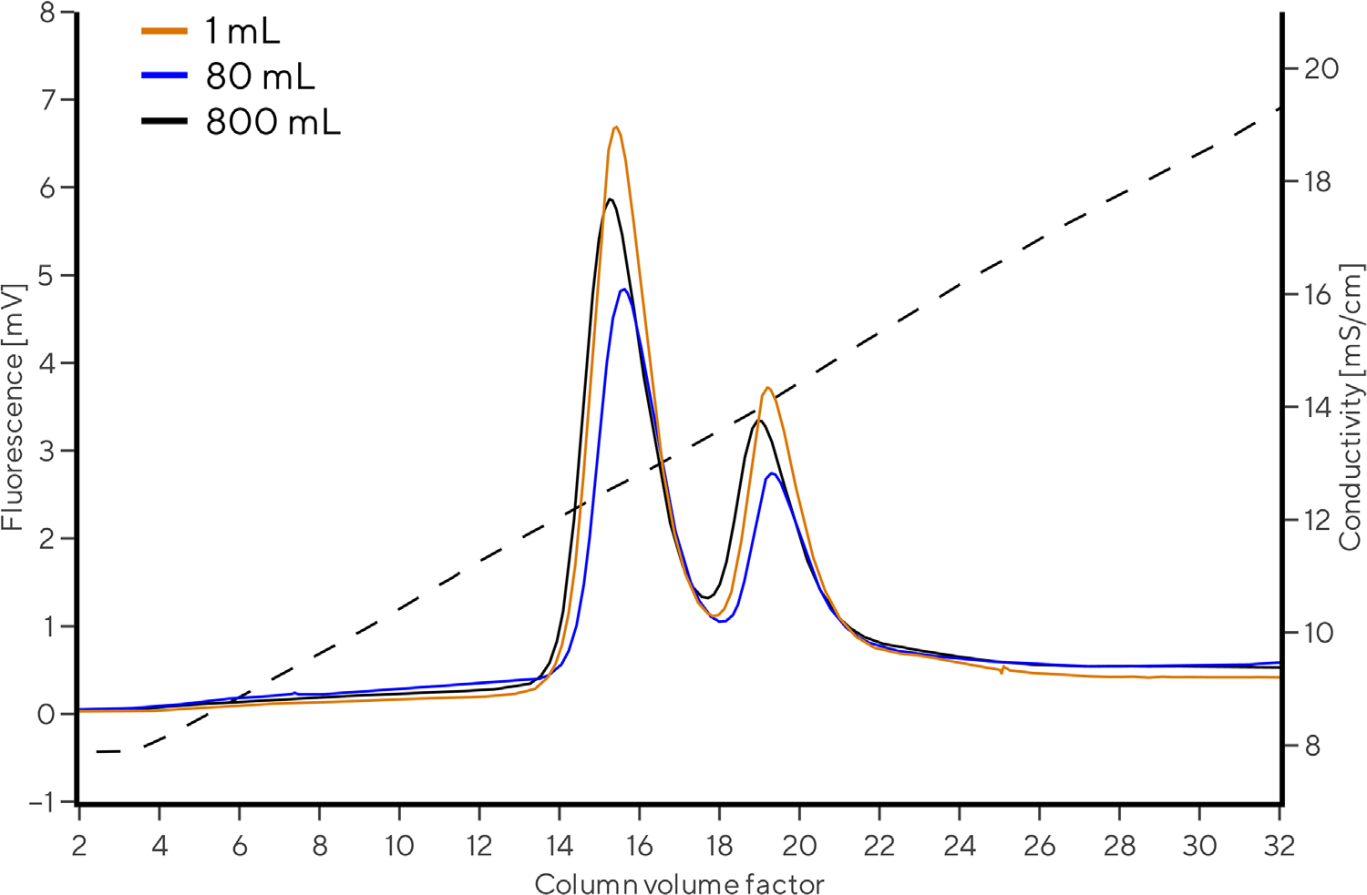
Chromatograms demonstrating elution of rAAV2/8 empty and full capsids on CIMmultus QA 1 mL (red line), 80 mL (blue line), and 800 mL (black line) columns. For visual comparison, the chromatograms are normalized to column volume factor and adjusted for gradient start.

Results from Figure 4 and Table 1 demonstrated comparable rAAV2/8 elution profile for 1, 80, and 800 mL CIMmultus QA columns. *[KCl]*_empty_ ranged between 90.4 and 91.4 mM, *[KCl]*_full_ between 102.5 and 104.2 mM and *R*_full/empty_ capsids between 1.18 and 1.35. All column sizes efficiently and similarly separated empty and full rAAV2/8 capsids. This confirmed the possibility of efficiently scaling up the CIMmultus QA chromatographic column line for the E/F AAV applications.

**TABLE 1:** KCl concentration at empty and full capsid elution and E/F resolution for the scale‐to‐scale E/F rAAV2/8 separation experiment.

	1 mL column	80 mL column	800 mL column
*[KCl]*_empty_ (mM)	90.4	91.4	90.4
*[KCl]*_full_ (mM)	103.5	104.2	102.5
*R*_full/empty_	1.35	1.21	1.18

### 3.3 Correlation Between Ionic Capacity and Elution of E/F rAAV2/8 Capsids

AAV separation is neither a practical nor a cheap experiment for evaluation of large‐scale chromatographic columns, therefore other chromatographic methods were evaluated for CIM QA columns and correlated with E/F AAV results.

Ionic capacity (pH transients) is a fast, simple, cheap and non‐destructive method for determining the amount of available charged groups on ion‐exchange chromatographic monoliths [36]. It is already used as one of quality control release testing methods for CIM monolithic columns. Ionic capacity of ion exchangers correlates with the total amount of ion‐exchange groups accessible to small ions, such as phosphates.

**FIGURE 5:**
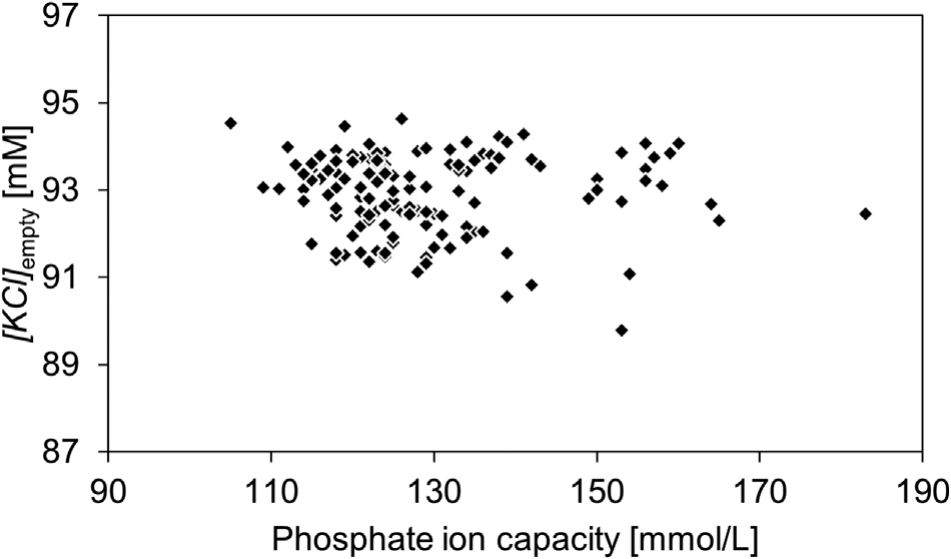
Correlation between *[KCl]*_empty_ and phosphate ion capacity for CIMmultus QA 1 mL columns.

The same columns shown in Figure 3 were additionally evaluated for phosphate ion capacity. The correlation between *[KCl]*_empty_ and ionic capacity is shown in Figure 5. Columns with a broad range of phosphate ion capacity between 110 up to 190 mmol/L (this corresponds to a range of 1.1 up to 1.8 mmol QA groups per mL of support, according to Figure 7 in [36]) eluted empty rAAV2/8 capsids at similar *[KCl]*. The density of charges on the surface of chromatographic material is obviously not the only parameter affecting the adsorption/desorption of AAV particles.

Several other methods utilizing simple organic chromatographic probes of different sizes (ethyl acetate, glutathione, lysozyme, etc.) were additionally evaluated, but none of them could predict salt concentration at AAV elution. The interaction of AAV particles with the surface is too complex to be described by the elution pattern of simpler and smaller probes. Therefore, AAV separation itself was selected as the only viable option for characterizing QA chromatographic columns, intended for reproducible AAV purification.

### 3.4 Implementing 0.2 mL Specimen Columns and Their Chromatographic Testing

The decision was made to implement E/F rAAV2/8 capsid separation as a product release test method and for comparing the performance of QA monolithic columns from different batches and scales (from 1 mL to 8 L bed volume). Two crucial requirements needed to be fulfilled first: (1) having complete control over the AAV separation method; (2) robustly obtaining representative small‐scale monolith columns from large monoliths. The robust E/F rAAV2/8 separation method was successfully developed, as described in Section 3.1. For the second requirement, the technology of obtaining 0.2 mL disc‐shaped samples (specimens) from the original large‐scale monolith was developed and optimized (for 40 mL bed volume and above). The procedure is described in Section 2.4.1. The decision for a small column volume was made to minimize rAAV2/8 sample consumption during testing and accelerate evaluation time. 0.2 mL column volume was selected as optimal, as further decreasing column size would result in an excessive influence of the chromatographic system's dead volume on the AAV separation. One difference between parental and specimen columns is radial versus axial flow distribution, but it was not considered as a critical parameter due to flow rate independent separation of biomolecules on convection‐based chromatographic supports, including monoliths [41].

The method for E/F rAAV2/8 separation was downscaled from CIMmultus QA 1 mL column (radial format) to 0.2 mL specimen columns (axial format) using the iso‐conductivity approach [37]. 0.2 mL is a small bed volume format and as such more prone to be influenced by variability of the LC system characteristics. Validating the chromatographic method on this column format was therefore a crucial next step.

The robustness of the method was increased by precise buffer preparation, controlling temperature during testing, evaluating the results correctly, and running a reference column before testing. The most reproducible and precise buffer preparation was achieved when pH of the buffer was adjusted by adding a fixed amount of standardized 1 M HCl (Titripur). This buffer preparation procedure enabled us to prepare buffers within not more than ± 0.05 pH difference and not more than ± 0.1 mS/cm and ± 0.2 mS/cm difference in conductivities of buffers A and B, respectively. This ensured highly reproducible AAV separation with different sets of buffers. Temperature affects the retention times of AAV capsids. Therefore, using a thermostat at 23 ± 1°C was essential to ensure consistent and repeatable column performance. The E/F rAAV2/8 separation method on specimen columns (described in Section 2.4.5) was validated using one specimen column, obtained from an 800 mL monolith (Table 2).

**TABLE 2:** Intra‐day, inter‐day and PATfix repeatability of E/F rAAV2/8 separation on 0.2 mL QA specimen columns. Average value, standard deviation (*σ*) and relative standard deviation (RSD) are shown for *[KCl]*_empty_ and *R*_full/empty_.

		*[KCl]*_empty_	*R*_full/empty_
Intra‐day repeatability	Average (*n* = 6)	91.70 mM	0.843
	*σ*	0.18 mM	0.005
	RSD	0.2%	0.6%
Inter‐day repeatability	Average (*n* = 12)	91.66 mM	0.840
	*σ*	0.17 mM	0.014
	RSD	0.2%	1.7%
Repeatability on three PATfix systems	Average (*n* = 9)	92.90 mM	0.764
	*σ*	0.40 mM	0.038
	RSD	0.4%	5.0%

Additionally, one specimen obtained from an 800 mL monolith was tested over a period of 11 months to determine the long‐term repeatability of the separation method. Forty‐six injections (one on each test day) were performed, where the average *[KCl]*_empty_ was 92.4 mM with a *σ* of 0.8 mM (0.9% RSD). The average E/F resolution was 0.80 with a *σ* of 0.12 (15% RSD). Measurement uncertainty obtained from this experiment includes the effect of buffer preparation, different sample aliquots, system operator, and variability in system and column performance. As mentioned previously, resolution is scattering more profoundly than *[KCl]*_empty_ and these two parameters do not correlate.

Next, the repeatability of specimen preparation was evaluated by testing 10 specimen columns obtained from the same slice of an 800 mL monolith. The average for *[KCl]*_empty_ was 92.4 mM with a *σ* of 0.4 mM (0.4% RSD). The *σ* for repeatability of specimen preparation was twice higher than the intra‐day repeatability (Table 2), meaning there is some effect of specimen sampling on the results of E/F rAAV2/8 separation.

To estimate the total measurement uncertainty of the testing procedure from our data, we combined the variability in specimen preparation (already includes intra‐day repeatability) and variability in long‐term evaluation. At a coverage factor of 2, this adds up to a total measurement uncertainty of approximately ± 1.79 mM. To accommodate some potential small variability in material, our target range in *[KCl]*_empty_ for column reproducibility was set to 92.3 mM ± 3.0 mM KCl.

### 3.5 Homogeneity of 80, 800, and 8000 mL Monolithic Polymers

**FIGURE 6:**
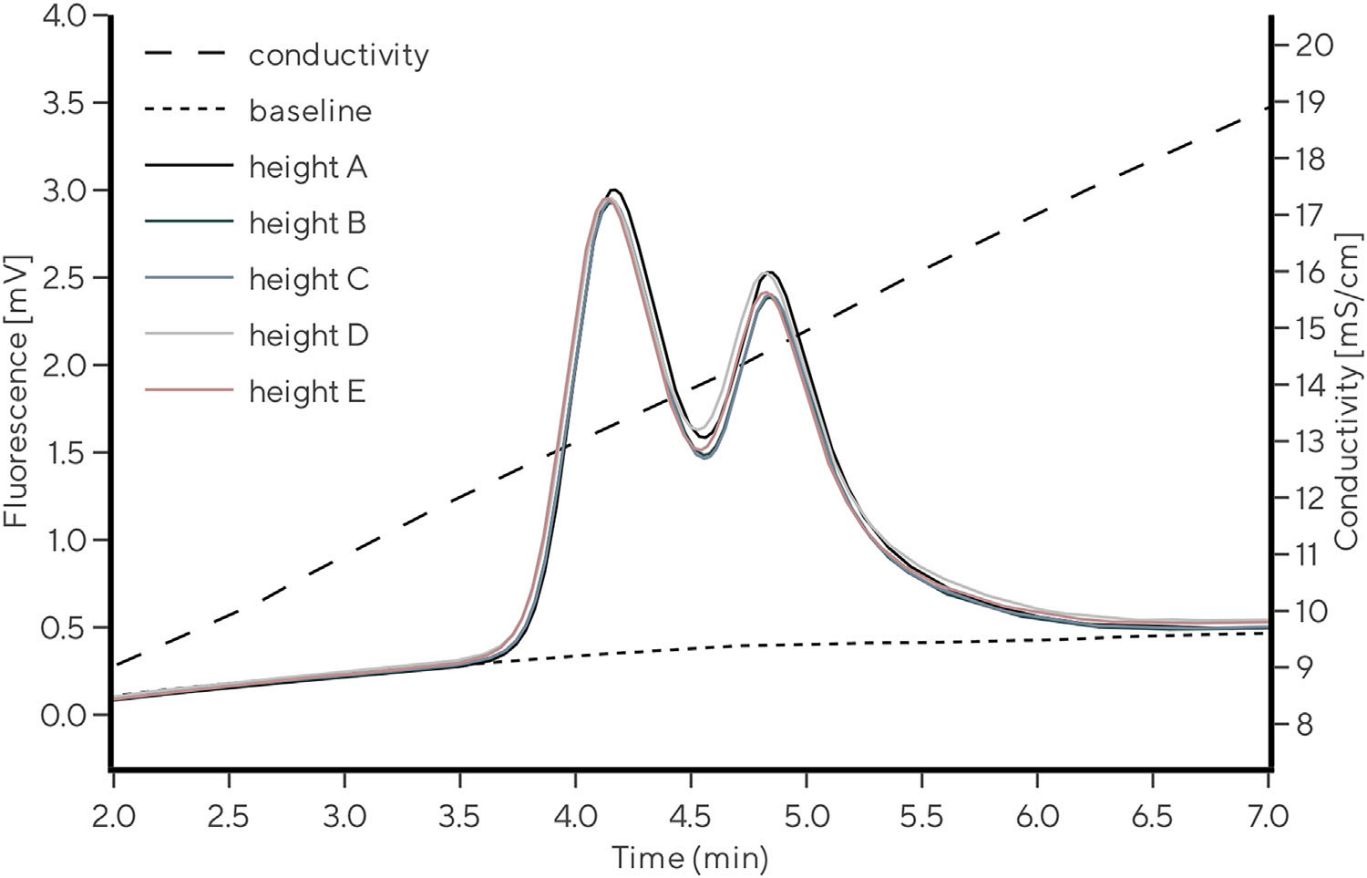
Chromatograms of E/F AAV separation representing vertical homogeneity of the 8000 mL CIMmultus QA monolith. A representative specimen (out of four) from each height is shown.

With the validated AAV separation method in hand the homogeneity of CIMmultus QA monoliths was evaluated by testing 20 specimen columns obtained at four horizontal positions (angles of 0°, 90°, 180°, and 270°) from five vertical positions (slices A–E) of parental monoliths (80, 800, and 8000 mL) according to the Figure S1. Figure 6 shows representative chromatograms of E/F rAAV2/8 capsid separation using specimen columns from five slices of an 8000 mL CIMmultus QA monolith, demonstrating its vertical and horizontal homogeneity.

The average *[KCl]*_empty_ for 20 specimens per monolith over the entire 80, 800, and 8000 mL QA monoliths were 93.4 mM (0.5% RSD), 93.5 mM (0.7% RSD) and 94.4. mM (0.5% RSD), respectively. This scattering is similar to scattering of specimen sampling from one single height (see Section 3.5), demonstrating excellent vertical homogeneity of the large‐scale monoliths. This further proves that polymerization and surface modification of the monoliths are homogeneous throughout the whole bed volume.

### 3.6 Matching Chromatographic Properties between Specimens and Parental Chromatographic Column

**FIGURE 7:**
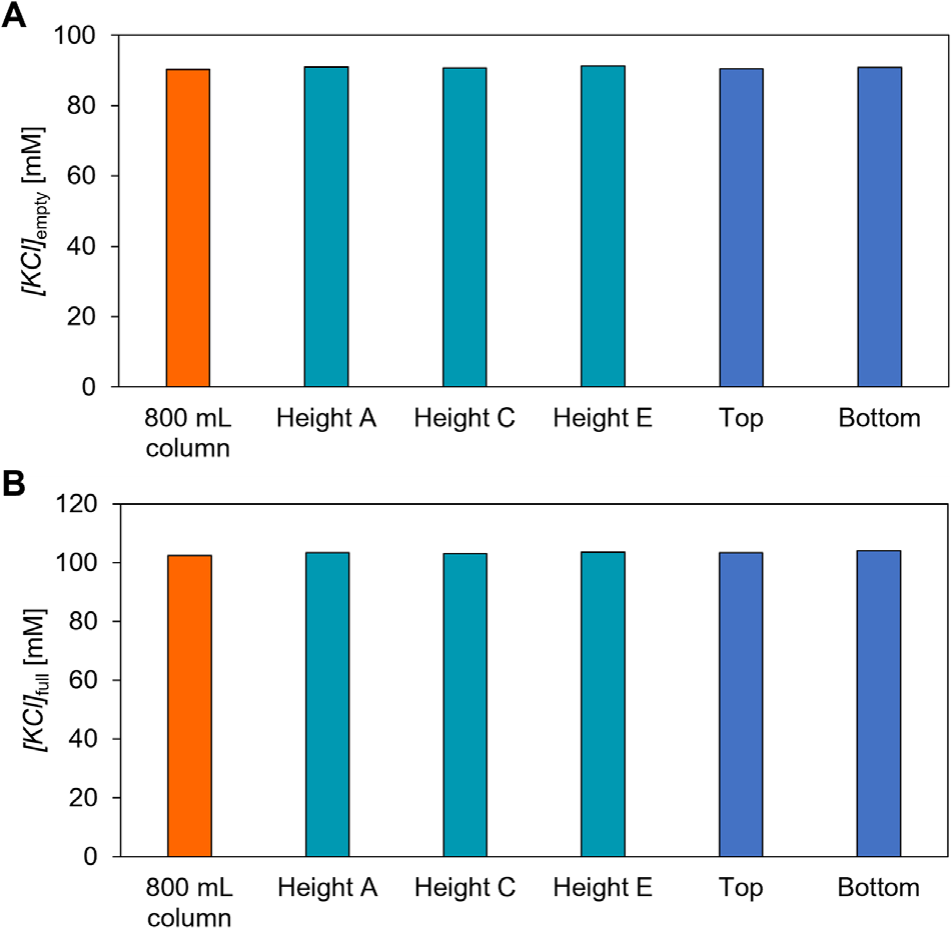
Separation of E/F rAAV2/8 capsids on whole 800 mL column, top and bottom specimen columns, and specimen columns from inside the packed column (heights A, C, and E). *[KCl]*_empty_ (A) and *[KCl]*_full_ (B) are shown.

Our next goal was to confirm that specimen columns match the chromatographic properties of their parental column. Specimen columns from the material above (top) or below (bottom) the packed 800 mL monolith were obtained (see Section 3.2) and both the parental column and its specimen columns were evaluated for E/F rAAV2/8 separation under the same conditions (sample, sample loading amount, gradient composition, temperature). Then, the 800 mL column was unpacked and the monolith was cut into vertical slices. Specimen columns were obtained from heights A, C, and E (see Figure S1) of the material, which was previously inside the evaluated parental column. The new specimen columns were again evaluated for E/F rAAV2/8 capsid separation under the same conditions as the parental column and specimens before. *[KCl]*_empty_ with 800 mL column and all the 0.2 mL specimen columns were in the range of 90.8 ± 0.3 mM (Figure 7), confirming the matching of chromatographic properties between large chromatographic units and specimen columns. This confirms that using specimen columns as test units will suitably predict the E/F AAV capsid separation properties of parental columns.

### 3.7 Batch‐to‐Batch and Scale‐to‐Scale Reproducibility of Chromatographic Monoliths

Column lot‐to‐lot reproducibility is crucial to achieve enhanced downstream process robustness, especially with such delicate separations as E/F AAV. Specimen columns could serve as a platform for testing each individual industrial chromatographic column prior to release, while maintaining cGMP‐compliance of the parental column. This would allow the selection of columns with very narrow acceptance criteria and provide the background for high‐reproducibility column lines.

**FIGURE 8:**
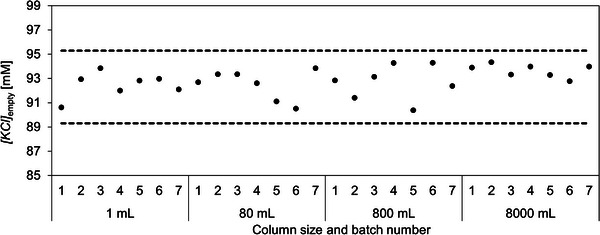
Separation of E/F rAAV2/8 capsids on CIMmultus QA 1 mL columns and specimens from CIMmultus QA 80, 800, and 8000 mL columns. 7 production batches of each size were evaluated. *[KCl]*_empty_ is plotted with circles, while dased line represents the target *[KCl]*_empty_ range of 92.3 ± 3 mM.

To evaluate batch‐to‐batch and scale‐to‐scale consistency of columns, E/F rAAV2/8 capsid separation was performed on 28 different monolith batches of CIMmultus QA columns, ranging from 1 mL up to 8000 mL bed volume. 0.2 mL specimen columns were used for testing the monolith batches above 80 mL bed volume, while 1 mL columns were evaluated intact. All column batches matched our desired *[KCl]*_empty_ range of 89.3–95.3 mM (Figure 8), established in Section 3.4. This confirmed excellent batch‐to‐batch and scale‐to‐scale consistency of CIMmultus QA columns. The achieved chromatographic reproducibility of QA columns served as the foundation for commercial CIMmultus QA HR chromatographic column line.

## 4 Conclusions

The primary objective of the study was to develop a testing procedure for CIM QA chromatographic monolithic columns, which aims to select columns that exhibit a highly reproducible E/F AAV elution pattern. Our focus was on identifying columns that maintain a consistent salt concentration during AAV elution, as this consistency facilitates robust step gradient separation of AAV capsids and allows for controllable industrial scaling. Initial experiments explored simple, non‐destructive chromatographic methods using small organic or inorganic probes. However, none of these methods and probes could accurately predict the salt concentration at elution of AAV particles. Therefore, AAV separation itself was selected as the only viable option for characterizing QA chromatographic columns, intended for reproducible AAV purification.

The elution patterns for the separation of E/F rAAV2/8 capsids on 1, 80, and 800 mL CIMmultus QA columns demonstrated consistent elution conditions, with empty capsids eluting at a KCl concentration range of 89.4–91.4 mM. Two critical requirements for implementation of this method were initially met: (1) excision of representative small‐scale (0.2 mL) monolith units, referred to as specimen columns, from larger monoliths, and (2) establishment of the E/F rAAV2/8 capsid separation method on specimens with a total measurement uncertainty of less than 3 mM in *[KCl]*_empty_. The scalability between the specimen and the parental column was confirmed for the 800 mL monolith. The *[KCl]*_empty_ for both the 800 mL column and the corresponding 0.2 mL specimens was within the range of 90.8 ± 0.3 mM. These results enabled the use of specimens as test units for parental columns.

The intra‐column monolith homogeneity of 80, 800, and 8000 mL monoliths, determined through average *[KCl]*_empty_ for 20 specimens per monolith, was 93.4 mM (0.5% RSD), 93.5 mM (0.7% RSD) and 94.4. mM (0.5% RSD), respectively. A uniform polymerization and surface modification of the monolith across the entire bed volume was demonstrated.

Finally, the validated E/F rAAV2/8 capsid separation method on specimens and 1 mL columns was implemented to monitor a larger number of CIMmultus QA production batches (bed volumes of 1–8000 mL). The elution of empty rAAV2/8 capsids at KCl concentrations between 89.3 and 95.3 mM confirmed the robustness of CIM QA manufacturing and validated the scale‐to‐scale and batch‐to‐batch reproducibility of the columns. The new tools—specimen technology and robust AAV separation method—were the basis for the establishment of a novel industrial column production line, named the CIMmultus QA high reproducibility (HR) line, primarily intended for reproducibile separation of AAVs. Our next goal is to develop a scalable and manufacturing‐compatible downstream processing strategy for the enrichment of F AAV capsids using the new column line.

## Author Contributions

**Rok Miklavčič**: investigation, methodology, writing–original draft. **Tina Simčič**: conceptualization, methodology, writing–original draft, supervision. **Sara Rotar**: methodology, investigation. **Polona Komel**: investigation. **Rok Žigon**: conceptualization, methodology, writing–review & editing. **Dona Pavlovič**: conceptualization, methodology. **Ines Bergoč**: conceptualization, methodology. **Domen Ipavec**: investigation. **Ana Simčič Zuljan**: investigation. **Ažbe Žnidaršič**: writing–review & editing, supervision. **Dolores Kukanja**: methodology, supervision. **Jana Vidič**: methodology, supervision. **Aleš Štrancar**: funding acquisition, writing–review & editing, supervision. **Urh Černigoj**: conceptualization, methodology, writing–original draft, supervision.

## Conflicts of Interest

All authors were employed by the company Sartorius BIA Separations d.o.o. The columns and LC instruments used in this research were paid for and made available by Sartorius BIA Separations d.o.o.

## Supporting information



Supporting Information

## Data Availability

The data that support the findings of this study are available from the corresponding author upon reasonable request.
