# Supplementary material for: Development and Validation of AAV Capsids Separation on Specimen Columns for Reproducibility Evaluation of Large‐Scale Chromatographic Monoliths
Source: J Sep Sci. 2025 Mar 17;48(3):e70114. doi: 10.1002/jssc.70114 (PMC11914862; doi:10.1002/jssc.70114)

**Supplementary material**

**Development and validation of AAV capsids separation on specimen columns for reproducibility evaluation of large-scale chromatographic monoliths**

Rok Miklavčič^1,2^, Tina Simčič^1^, Sara Rotar^1^, Polona Komel^1^, Rok Žigon^1^, Dona Pavlovič^1^, Ines Bergoč^1^, Domen Ipavec^1^, Ana Simčič Zuljan^1^, Ažbe Žnidaršič^1^, Dolores Kukanja^1^, Jana Vidič^1^, Aleš Štrancar^1^, Urh Černigoj^1,^*

1 Sartorius BIA Separations d.o.o., Mirce 21, 5270 Ajdovščina, Slovenia

2 Faculty of Medicine, University of Ljubljana, Vrazov trg 2, 1000 Ljubljana, Slovenia

*** Corresponding author**:

Urh Černigoj

Sartorius BIA Separations d.o.o., Mirce 21, 5270 Ajdovščina, Slovenia

E-mail address: [urh.cernigoj@biaseparations.com](mailto:urh.cernigoj@biaseparations.com)

**Table of contents**

**Figure S1.** Schematic representation of vertical positions, from which specimen columns were obtained during the evaluation

**Figure S2.** Schematic representation of system configuration for separation of E/F rAAV2/8 capsids using 80 mL and 800 mL columns.

**Figure S1.** Schematic representation of vertical positions, from which specimen columns were obtained during the evaluation. Typically, specimen columns from top and bottom position were analysed. Positions A-E are located inside the packed parental column.


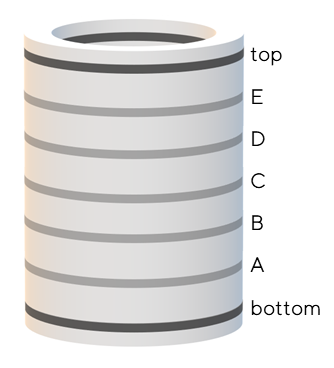


**Figure S2.** Schematic representation of system configuration for separation of E/F rAAV2/8 capsids using 80 mL and 800 mL columns. A splitting tee was used to split the flow path into two lines – the main (thick line) and detector line (thin line). Signals were monitored on the detector line with a conductivity monitor and a fluorescence detector. Flow rate through this line was regulated to be around 1 mL/min by optimizing the length of capillary tubing after the detectors.


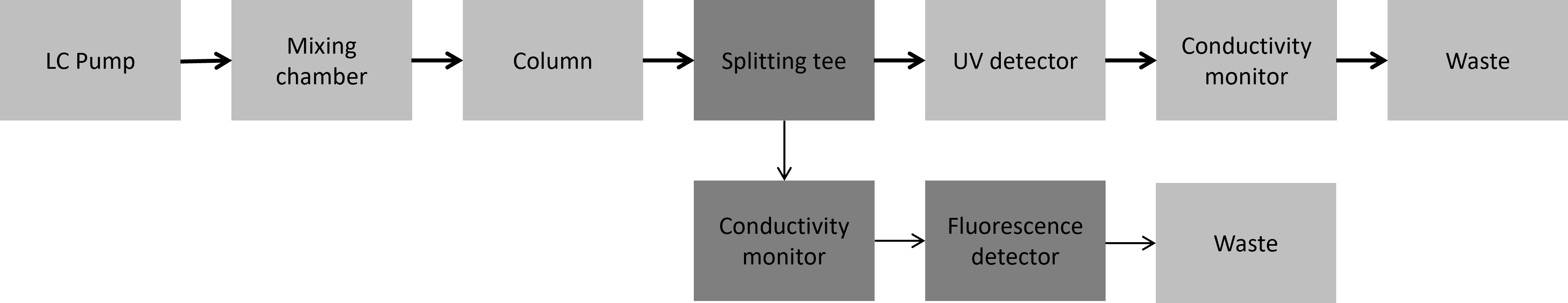

Supplement: Supplementary file 1 — Supporting Information [file JSSC-48-e70114-s001.docx]
